# *Herpes labialis*: a rare clinical image

**DOI:** 10.11604/pamj.2022.41.325.34635

**Published:** 2022-04-22

**Authors:** Mayur Bhaskar Wanjari, Tejaswee Lohakare

**Affiliations:** 1 Department of Research and Development, Jawaharlal Nehru Medical College, Datta Meghe Institute of Medical Sciences, Sawangi, Wardha, Maharashtra, India,; 2 Department of Child Health Nursing, Srimati Radhikabai Meghe Memorial College of Nursing, Datta Meghe Institute of Medical Sciences, Sawangi, Wardha, Maharashtra, India

**Keywords:** *: Herpes labialis*, mucous membrane, sore throat, fever

## Image in medicine

*Herpes labialis* is a rash of the skin and mucous membranes (particularly the lips) and is characterized by erythema and blisters that are preceded and accompanied by burning pain. We present a case of a 21-year-old male who comes to the emergency department with a complaint of sore throat and fever for ten days. On the patient´s physical examination, the upper and lower lips showed redness with a white patch, and histopathological examination suggested *herpes labialis*. The patient was referred to the dermatological department for further management.

**Figure 1:**
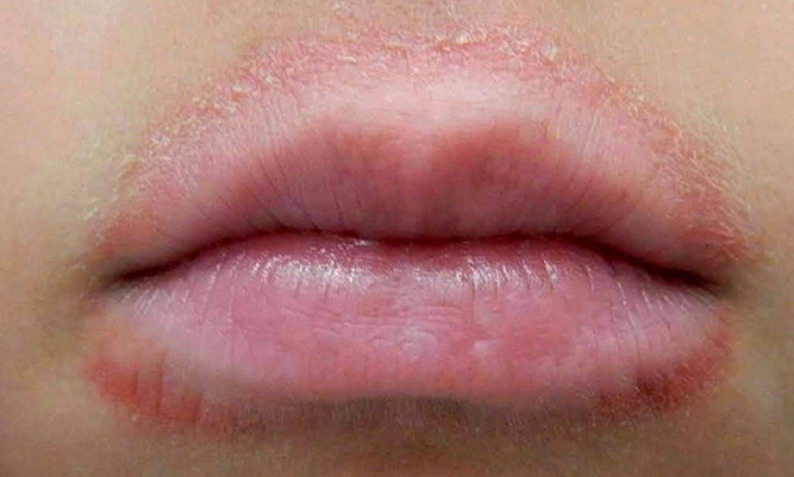
upper and lower lips are seen redness with a white patch

